# The HB22.7 Anti-CD22 monoclonal antibody enhances bortezomib-mediated lymphomacidal activity in a sequence dependent manner

**DOI:** 10.1186/1756-8722-4-49

**Published:** 2011-12-01

**Authors:** Shiloh M Martin, Eric Churchill, Hayes McKnight, Christopher M Mahaffey, Yunpeng Ma, Robert T O'Donnell, Joseph M Tuscano

**Affiliations:** 1 Department of Internal Medicine, Division of Hematology and Oncology, University of California, Davis Cancer Center, (4501 X Street), Sacramento, CA (95817), USA; 2 Millennium Pharmaceuticals Inc., (40 Landsdown St Cambridge MA (02139) USA; 3 Northern California Veterans Administration Healthcare System, (10535 Hospital Way), Mather, CA (95655), USA

**Keywords:** HB22.7, CD22, bortezomib, Velcade, proteasome inhibition, Non-Hodgkin's lymphoma, reactive oxygen species, apoptosis, mantle cell lymphoma

## Abstract

Most non-Hodgkin's lymphomas (NHL) initially respond to chemotherapy, but relapse is common and treatment is often limited by chemotherapy-related toxicity. Bortezomib, is a highly selective proteasome inhibitor with anti-NHL activity; it is currently FDA approved for second-line treatment of mantle cell lymphoma (MCL). Bortezomib exerts its activity in part through the generation of reactive oxygen species (ROS) and also by the induction of apoptosis.

We previously validated CD22 as a potential target in treating NHL and have shown that the anti-CD22 ligand blocking antibody, HB22.7, has significant independent lymphomacidal properties in NHL xenograft models. We sought to determine whether or not these agents would work synergistically to enhance cytotoxicity. Our results indicate that treatment of NHL cell lines with HB22.7 six hours prior to bortezomib significantly diminished cell viability. These effects were not seen when the agents were administered alone or when bortezomib was administered prior to HB22.7. Additionally, HB22.7 treatment prior to bortezomib increased apoptosis in part through enhanced ROS generation. Finally, in a mouse xenograft model, administration of HB22.7 followed 24 hours later by bortezomib resulted in 23% smaller tumor volumes and 20% enhanced survival compared to treatment with the reverse sequence. Despite the increased efficacy of HB22.7 treatment followed by bortezomib, there was no corresponding decrease in peripheral blood cell counts, indicating no increase in toxicity. Our results suggest that pre-treatment with HB22.7 increases bortezomib cytotoxicity, in part through increased reactive oxygen species and apoptosis, and that this sequential treatment combination has robust efficacy *in vivo*.

## Introduction

Non-Hodgkin's lymphomas (NHL) are a heterogeneous group of lymphoid malignancies; the majority are of B-cell origin [1]. Incidence rates have almost doubled in the last 40 years and NHL is now the sixth most common cause of cancer-related death in the US [2]. Initial therapy for NHL includes chemotherapy, biologic therapy, and radiotherapy, but relapse is common and the efficacy of chemotherapy is limited by toxicity [1]. Therefore, novel, less toxic therapeutic combinations are needed to improve patient survival.

Bortezomib (Velcade, PS-341) is a reversible inhibitor of the 26S proteasome [3] and is approved for the treatment of multiple myeloma and relapsed mantle cell lymphoma. The mechanism by which bortezomib induces apoptosis is not completely understood, but is thought to involve the accumulation of NF-kB [3,4], increased ROS generation [5,6], and activation of the unfolded protein response [7,8]. Bortezomib has shown robust preclinical anti-tumor activity in several NHL cell lines including MCL, FL and Burkitt's lymphoma [9,10]. Five independent studies led to the approval of bortezomib by the FDA as second line treatment of MCL [11-15] and its efficacy in FL has been studied in phase I trials [3]. Additional phase II [11,14,16] and phase III studies in FL are ongoing.

As B-lymphocytes mature to fully differentiated plasma cells, the B-lymphocyte-specific glycoprotein, CD22, which is expressed by nearly all mature B-lymphocytes, disappears [17]. The two amino-terminal immunoglobulin (Ig) domains of CD22 mediate ligand binding and hetero- and homotypic cell adhesion [18-20] and studies have demonstrated that the ligand binding domains are critical for B-cell receptor signaling and B-cell survival [21]. MAbs such as HB22.7, which target these amino terminal Ig domains and block the interaction of CD22 with its ligand, are effective at inducing proliferative responses in primary B-cells while activating apoptotic pathways in neoplastic B-cells [22]. Since most NHLs express CD22, this glycoprotein is a promising target for immunotherapy. We previously reported the lymphomacidal properties of HB22.7 in nude mice bearing Raji (human B-cell NHL) xenografts [22].

Because of bortezomib's pronounced cytotoxic effects and unique mechanism of action, novel agents in NHL are increasingly being studied in combination with bortezomib [23-26]. In preclinical studies, additive cytotoxic effects have been reported with the combination of bortezomib and the anti-CD20 mAb rituximab (Rituxan) in B-cell lymphoblastic leukemia (B-CLL) and MCL [25-27]. The combination has been found to be active in a Phase II clinical trial [16] and is now being compared to single arm rituximab in a Phase III trial in relapsed FL. The cytotoxic effect of rituximab occurs via multiple pathways, one of which is the downregulation of the anti-apoptotic protein Bcl-xL [28] and in B-NHL cell lines, Bcl-xL down-regulation occurs partly via inhibition of NF-kB activation [29]. Interestingly, crosslinking CD22 with HB22.7 can similarly down regulate Bcl-xL [20]. Since proteasome inhibition by bortezomib also inhibits NF-kB activation [3,4], which in turn modulates levels of Bcl-2 family members such as Bcl-xL [5,30,31], this suggests that the combination of HB22.7 and bortezomib may be additive. Additionally, studies have shown that some of rituximab's cytotoxic effects are complement mediated, occurring through ROS generation [32]. In addition to its effects on NF-kB, bortezomib increases ROS generation [5,6]. The effect of HB22.7 on ROS production has not been previously determined. However since rituximab and bortezomib enhance cytotoxicity in part through ROS generation and NF-kB inhibition and HB22.7 cross-linking of CD22 can similarly downregulate Bcl-xL, we hypothesized that HB22.7 may also exhibit enhanced cytotoxicity against malignant B-cells when combined with bortezomib, in part through increased ROS generation.

To determine this, we used both *in vitro *cell culture and *in vivo *mouse xenograft NHL models to determine the effects of HB22.7 or bortezomib treatment alone and in combination (concurrently and sequentially), on cytotoxicity, apoptosis, ROS induction, tumor volume, and survival.

## Materials and methods

### 1. Materials

RPMI 1640 medium, penicillin-streptomycin, fetal bovine serum (FBS) and 5-and 6-carboxy-2', 7'-dichlorodihydrofluorescein diacetate (carboxy-H_2_DCFDA) mixed isomers were purchased from Invitrogen/Life Technologies (Carlsbad, CA). WST-1 proliferation reagent was purchased from Roche (Indianapolis, IN). The mouse anti-human CD22 mAb, HB22.7, was purified from ascites and has been previously characterized [20]. Bortezomib was obtained from Millennium Pharmaceuticals (Cambridge, MA). All chemicals were of analytical grade purity.

### 2. Cell lines

The human Burkitt's B-cell lymphoma lines, Raji (CCL-86) and Ramos (CRL-1596), and the mantle cell lymphoma line, Granta-519 (ACC-342) were obtained from American Type Culture Collection (Rockville, MD). The cells were grown in suspension in full RPMI (supplemented with 10% FBS, 50 units/ml penicillin G, and 50 μg/ml streptomycin sulfate). The cells were maintained in tissue culture flasks at 37°C in 5% CO_2 _and 90% humidity. Cells used for experiments were harvested while in the log growth phase.

### 3. *In vitro *cytotoxicity assays

**Figure 1:**
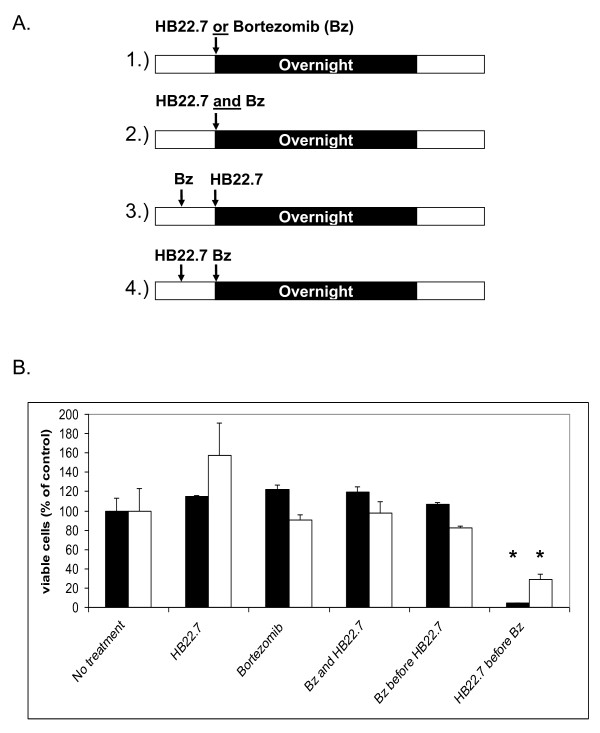
**Cells treated with HB22.7 followed by bortezomib demonstrate increased cytotoxicity**. A. Ramos or Granta-519 cells were treated overnight with either bortezomib (Bz) (75 nM) or HB22.7 (60 μg/ml) alone (1]), bortezomib and HB22.7 concurrently (2]), bortezomib six hours prior to overnight treatment with HB22.7 (3]), or HB22.7 six hours prior to bortezomib overnight treatment (4]). B. Viability of Ramos (black bars) or Granta-519 (white bars) were measured spectrophotometrically utilizing the dye WST-1 and is expressed as % control (untreated) cells. Treatments are listed on the X-axis. Error bars = SEM. (*) = p-value < 0.05 against all other treatment groups (n = 3).

Ramos or Granta-519 cells (5 × 10^4^/mL) were plated in 96 well flat bottom plates in a final volume of 100 uL. Cells were treated with bortezomib (75 nM) alone, HB22.7 (60 μg/mL) alone, bortezomib plus HB22.7, bortezomib followed 6 h later by HB22.7, or HB22.7 followed 6 h later by bortezomib (see Figure 1). Control cells received no treatment. Suboptimal doses (doses lower than those needed to cause cytotoxicity) of bortezomib and HB22.7 were used in order to detect additive or synergistic effects of combination treatment. All samples were plated in triplicate. The plates were then incubated at 37^°^C, 5% CO_2 _and 90% humidity overnight. After overnight treatment, WST-1 reagent was added (20 uL per well) and incubated for 2 h, after which the plate was read at 450 nm on an EMax precision microplate reader using SoftMax Pro software (Molecular Devices, Sunnyvale, CA). Absorbance readings were converted to % of control (untreated cells) and plotted.

### 4. ROS assay

Ramos cells (5 × 10^4^/mL) were seeded into T-25 flasks (5 mL per flask) and treated with bortezomib alone (15 μM), HB22.7 (100 μg/mL) alone, both agents concurrently, or one agent followed 6 h later by the second agent. Doses were chosen based on concentrations needed to cause cell death in this cell line in previous cell viability assays. All flasks were then incubated at 37°, 5% CO_2_, 90% humidity overnight. The next morning, cells were washed twice and resuspended in 5 mL RPMI-1640 without phenol red. Cells were then labeled with carboxy-H_2_DCFDA (final concentration 4.7 μM) for 90 minutes at 37°C, 5% CO_2_, 90% humidity. Cells were washed twice, then resuspended in 5 mL RPMI-1640 without phenol red and allowed to rest for 1 h at room temperature in the dark. Cells were then acquired on a flow cytometer (BD FACSCaliber, San Jose, CA) using the FL-1 parameter and analysis was performed using BD CellQuest software (San Jose, CA). The assay was repeated 3 times and the mean fluorescence intensity (MFI) was determined for each treatment group. The average fold increase in MFI over control (untreated cells) was calculated and plotted. Hydrogen peroxide was used as a positive control.

### 5. Apoptosis assay

Ramos cells (5 × 10^4^/mL) were seeded into T-25 flasks (5 mL per flask) and treated with bortezomib alone (15 μM), HB22.7 alone (100 μg/mL), both agents concurrently, or one agent followed 6 h later by the second agent. Doses were chosen based on concentrations needed to cause cell death in this cell line in previous cell viability assays. All flasks were then incubated at 37°, 5% CO_2_, 90% humidity for 24 h. After 24 h, cells were washed three times with PBS supplemented with 0.2% FBS and resuspended in 3 mL of PBS supplemented with 0.2% FBS containing 5 mg/mL propidium iodide. The cell samples were then acquired on a FACSCaliber flow cytometer using FL2-A and FL2-W parameters. Cell cycle analysis was performed using Verity ModFIT software (Topsham, ME) and the percentage of cells in the sub-G1 (apoptotic) fraction determined.

### 6. Mice and Xenograft model

Female athymic Balb/c nude (nu/nu) mice (Harlan Sprague Dawley, Indianapolis, IN) were housed and maintained according to University of California, Davis animal care guidelines. Raji cells were harvested in log growth phase and each mouse was injected subcutaneously with 0.5 × 10^6 ^cells on the left flank. The Raji cell line was used for xenografts rather than the Ramos cell line because Ramos xenografts tend to grow very rapidly, quickly becoming necrotic and therefore, resistant to uptake of treatment. After tumors were palpable, mice were divided into 4 groups (n = 5 per group) and treated with either bortezomib (20 μg) alone, bortezomib followed 24 h later by HB22.7 (2.1 mg), or HB22.7 followed 24 h later by bortezomib. Control mice were treated with PBS at volumes equivalent to mice receiving both bortezomib and HB22.7. All groups were treated twice weekly for two weeks (for a total of 4 treatments) and all treatments were administered via the tail vein. Tumor size was assessed twice weekly by measurement with calipers and tumor volume was calculated using the equation length × width × depth × 0.52. Blood samples were collected (n = 2 mice per treatment group) at days 0, 2, 5, 9, 12, 15, 18, 23, 26, 29, 33, 36, and 40 by nicking the tail vein. For red blood cell and platelet counts, 10 μl blood was diluted into 2 ml PBS pH 7.4 containing 0.5 M EDTA. For white blood cell counts, 20 μl blood was added to 380 μl of 2% acetic acid/1% crystal violet solution. All cells were counted on a hemocytometer.

### 7. Statistics

For *in vitro *data, individual groups were compared against each other using a two-tailed Student's t-test. For xenograft tumor volume data, individual mice in each treatment group were either ranked as 0 (did not achieve event) or 1 (achieved event). An event was defined as tumor volume reaching 450 mm^3 ^or greater. The time to event (in days) was then determined. If the event was not reached (ranked 0), a time to event of 84 days (the end of the study) was used. Treatment groups were then plotted against each other as Kaplan-Meier curves and the Log-rank test applied to determine Chi^2 ^and p-values. Results were considered statistically significant if calculated p-value was < 0.05. All statistical calculations were performed using GraphPad Prism software (San Diego, CA).

## Results and discussion

Additive cytotoxic effects have been reported with combination bortezomib/rituximab treatment in B-CLL and MCL [25,26] and the combination is currently being compared to single arm rituximab in a Phase III trial in relapsed FL, however the effects of sequencing these agents have not been fully explored. In terms of rationale, bortezomib and rituximab combination therapy allows for the targeting of the same pathways, such as NF-kB/Bcl-xL and ROS generation, by two different agents, potentially preventing resistance to either single agent alone. In choosing to explore the bortezomib/HB22.7 combination, we used a similar rationale that HB22.7's combination with bortezomib would result in alteration of apoptotic pathways, such as Bcl-xL, perhaps through enhanced ROS generation, ultimately leading to an increase in cytotoxicity and apoptosis in malignant B-cells.

In combination therapies, the sequence of treatment may affect the outcome. As reviewed by Shah and Schwartz, this sequence dependence can have multiple explanations, including, but not limited to, treatment induced alterations of the cell cycle, or pharmacodynamic interactions between two or more agents [33]. Many studies have demonstrated that treatment sequence may augment or inhibit efficacy in many types of cancer, both *in vitro *and *in vivo *[13,34-38]. In support of these studies, we previously demonstrated that HB22.7 had the greatest effects on NHL tumor volume shrinkage when administered simultaneously with and 24 hours after radioimmunotherapy [22]. Therefore, we examined both concurrent and sequential HB22.7 and bortezomib treatment approaches.

To determine if the combination of HB22.7 and bortezomib would produce additive or synergistic effects on cellular cytotoxicity, Ramos cells (Burkitt's B-cell NHL) or Granta-519 cells (MCL) were treated with each agent alone, both agents simultaneously or sequentially (treatment with one agent for 6 h followed by overnight treatment with the second agent) (Figure 1a). Suboptimal doses (doses lower than those needed to cause cytotoxicity by each drug individually) of bortezomib and HB22.7 were used to allow for detection of additive or synergistic effects of the combination. As seen in Figure 1b, treatment with HB22.7 alone, bortezomib alone, HB22.7 plus concurrent bortezomib, and bortezomib followed by HB22.7, had little to no cytotoxic effect. However, treatment with HB22.7 followed by bortezomib decreased the number of viable cells by about 95% (Figure 1b). This indicates that combination treatment with HB22.7 and bortezomib is synergistic and depends greatly on the sequence of treatment.

The lack of efficacy of either bortezomib or HB22.7 alone in the cytotoxicity assays was not surprising since we used suboptimal concentrations in order to determine if there was a synergistic effect of the two drugs together. However, we were surprised to see that the combination of the two drugs differed from our previous work which showed that the greatest efficacy was seen when HB22.7 was administered concurrently with or 24 hours after radioimmunotherapy [22]. At least in the case of the latter this may be explained by several reasons. By treating the cells with bortezomib first, the cells may be in cell cycle arrest before HB22.7 treatment has begun [39,40]. In effect, pretreatment with bortezomib may protect the cells from HB22.7's apoptotic actions. In addition, the accumulation of Mcl-1 caused by bortezomib treatment [27,41] may overwhelm HB22.7's ability to downregulate Mcl-1 [20]. A cleaved form of Mcl-1 in MCL cell lines treated with bortezomib has been reported [27] and it was shown that cleavage of Mcl-1 may affect its anti-apoptotic function [42]. Alinari et al suggest that a ratio of intact to cleaved Mcl-1 may be important in altering the apoptotic threshold [27]. Alternatively, proteasome inhibition may upregulate some factor which can act as a negative regulator of HB22.7's apoptotic effects.

**Figure 2:**
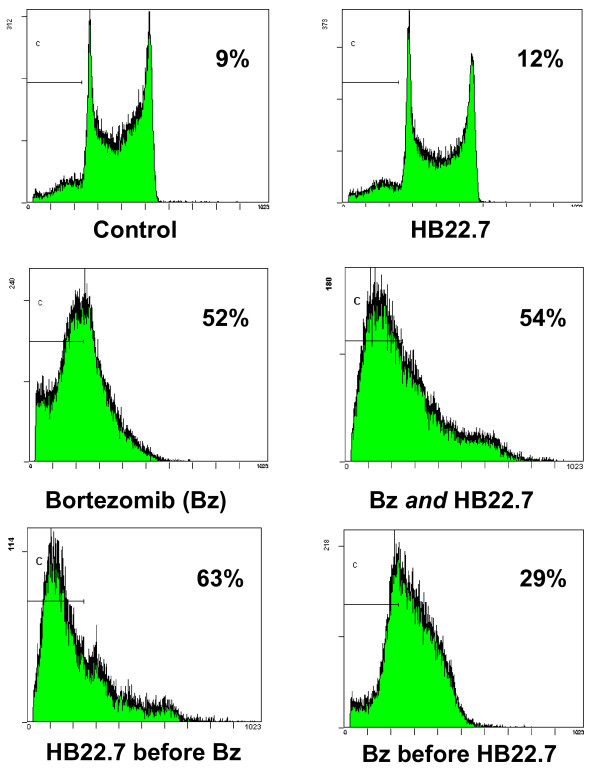
**Cells treated with HB22.7 followed by bortezomib demonstrate increased apoptosis**. Ramos cells were treated with bortezomib, HB22.7, or both (concurrently or sequentially) as described in Figure 1a and in Materials and Methods. Cells were stained with propidium iodide and apoptosis was assessed via flow cytometry by quantifying the sub-G1 (apoptotic) cell population. The percent of apoptotic cells is listed in the upper right corner of each panel. Results shown are representative of three separate experiments.

Using the Ramos cell line and same treatment paradigm outlined in Figure 1a, we next determined if this synergistic cytotoxicity was due to apoptosis. In support of the cell viability studies, concurrent addition of HB22.7 does little to improve the apoptotic effect of bortezomib (54% versus 52% for bortezomib alone), while the sequential treatment of HB22.7 followed by bortezomib enhances the apoptotic effect (63%), although this enhancement was not statistically significant (Figure 2). Interestingly, the reverse sequential treatment of bortezomib followed by HB22.7 actually induces less apoptosis (29%) than concurrent HB22.7/bortezomib (54%) or bortezomib alone (52%) (Figure 2).

**Figure 3:**
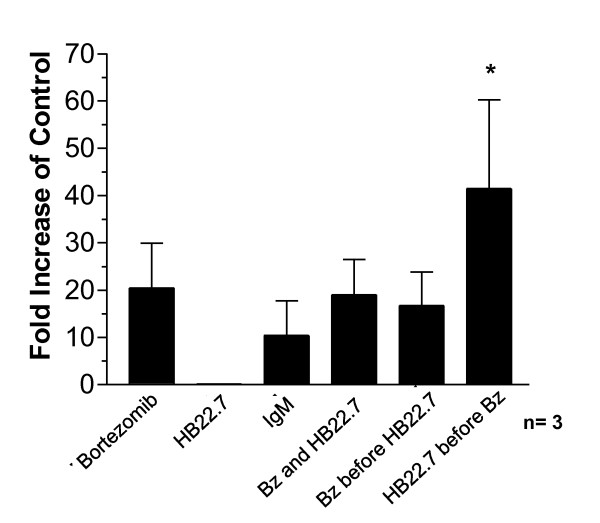
**Cells treated with HB22.7 followed by bortezomib demonstrate increased ROS generation**. Ramos cells were treated with hydrogen peroxide, bortezomib, HB22.7, or both bortezomib and HB22.7 (concurrently or sequentially) as shown in Figure 1a and described in Materials and Methods. Treatments are listed on the X-axis. ROS levels were assessed by flow cytometry and are presented as the fold increase in MFI (mean fluorescence intensity) compared to control (untreated) cells. Results shown are the average of three experiments. Error bars = SEM. (*) = p-value < 0.05 against all other treatment groups.

Since ROS generation has been shown to play an important role in bortezomib induced apoptosis [5,6] and in rituximab and anti-IgM induced B-cell death [32,43], we sought to determine if ROS levels were increased after HB22.7 treatment and if ROS generation might be enhanced in combination with bortezomib treatment. ROS generation in Ramos cells treated with the above mentioned protocols (Figure 1a) were examined. As a positive control, treatment of Ramos cells with hydrogen peroxide alone resulted in an expected increase in ROS production (3.5 ± 1.2 fold over control untreated cells) (data not shown). As shown in Figure 3, anti-IgM treatment, a known inducer of apoptosis in B-cell NHL [43], increased ROS by 10.4 ± 7.3 fold. In support of previous findings [5], bortezomib alone increased ROS generation by 20.4 ± 9.4 fold over untreated control cells (Figure 3). Interestingly, this did not correlate with increased cytotoxicity, which can be explained by the suboptimal concentrations of bortezomib used in the cell viability assays. The mechanisms of bortezomib induced cytotoxicity are thought to proceed through several different pathways and it is likely that while ROS levels are increased, other cytotoxic effects of bortezomib are not being initiated. Treatment with HB22.7 alone did not greatly induce ROS production (0.1 ± 9.8 × 10^-18 ^fold) and neither concurrent treatment with both agents nor treatment with bortezomib followed by HB22.7 elevated ROS beyond levels mediated by bortezomib alone (Figure 3). However, treatment with HB22.7 followed by bortezomib generated a robust 41.4 ± 18.8 fold increase in ROS over control untreated cells (Figure 3). Taken together, our *in vitro *data shows that the sequential combination of HB22.7 followed by bortezomib demonstrates synergistic cytotoxicity, and that this occurs via enhanced apoptosis and a synergistic increase in ROS generation.

**Figure 4:**
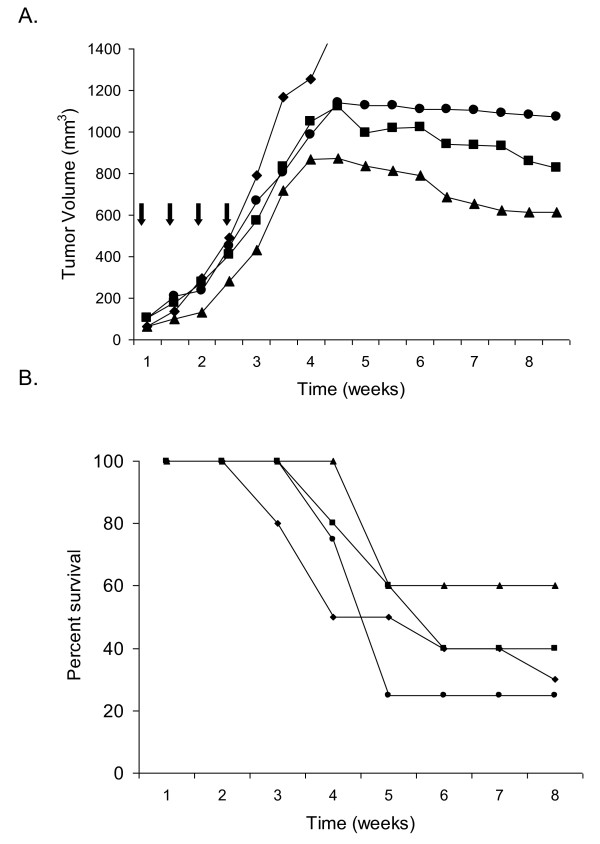
**Mice bearing NHL xenografts treated with HB22.7 treatment followed by bortezomib demonstrate reduced tumor volume and enhanced survival without increase in blood toxicity**. Nude mice (n = 5 per group) were implanted with Raji xenografts and treated twice weekly (indicated by arrows) with bortezomib alone (circle), bortezomib followed by HB22.7 (square), HB22.7 followed by bortezomib (triangle), or mock-treated (diamond) as shown in Figure 1a and described in Materials and Methods. Tumor volume was measured twice weekly as described in Materials and Methods. A) Tumor volume. B) Survival.

We next sought to determine if this *in vitro *synergy would translate to an *in vivo *mouse tumor xenograft model. Mice were implanted with Raji xenografts and treated with either bortezomib alone, or one agent followed 24 h later by the second agent as illustrated in Figure 1a and described in Materials and Methods. As shown in Figure 4a, mice that were treated with HB22.7 followed by bortezomib demonstrated 23.3% smaller tumor volumes than mice treated with the reverse sequence (bortezomib followed by HB22.7), 48.6% smaller tumor volumes than mice treated with bortezomib alone, and 62.8% smaller tumor volumes than control (mock-treated) mice. (Mean tumor volumes prior to treatment initiation and at the end of the 12 week study are listed in Table 1). Only the comparison between HB22.7 followed by bortezomib and control mice reached statistical significance (p-value < 0.05, Table 2). Mice treated with the reverse sequence (bortezomib followed by HB22.7) and bortezomib alone also had smaller tumor volumes (51.4% and 27.5%, respectively) than control mice, but these comparisons were not statistically significant (Table 2). It may be noted that tumors in all treatment groups grow in volume until about week 4, then appear to plateau. This is very typical of this xenograft model and even untreated tumors can sometimes plateau in volume once the tumor outgrows its blood supply and becomes necrotic. It is important to note however, that with all treatment arms the plateau occurs at a smaller tumor volume than in the untreated arm. While HB22.7 followed by bortezomib may not induce cure in these mice, it does demonstrate a statistically significant decreased tumor burden. In addition to smaller tumor volumes, mice treated with HB22.7 followed by bortezomib also demonstrated enhanced survival (60%) compared to mice treated with the reverse sequence (40%), bortezomib alone (25%), or control (30%) (Figure 4b). While a combination therapy may be more efficacious than single agent therapies, it is important to determine whether the combination increases toxicity. Toxicity was assessed by total body weight and peripheral blood cell counts of white blood cells, red blood cells, and platelets as described in Materials and Methods. In this xenograft model, mice treated with combination HB22.7/bortezomib demonstrated very little toxicity and no more than bortezomib alone, independent of how they were sequenced (data not shown). This indicates that while HB22.7 followed by bortezomib treatment demonstrates enhanced efficacy compared to the other treatment groups, there was no corresponding increase in toxicity.

**Table 1:** Change in tumor volume before and after treatment

Group	Volume Week 1 (mm^3^)	Volume Week 12 (mm^3^)	Volume change (mm^3^)
Control	65 ± 15.3	1447.7 ± 291.3	1382.7 ± 276
Bz alone	104 ± 6.6	1049.4 ± 381	945.4 ± 374.4
Bz → HB	104 ± 9.7	702.3 ± 260.3	598.3 ± 250.5
HB → Bz	64.6 ± 7.5	538.5 ± 281.5	473.9 ± 274.1

Mean tumor volumes ± SEM are listed for each group at weeks 1 and 12 of the study. Change in tumor volume between week 1 and week 12 are listed in the final column. The arrow denotes the order of treatment. Bz = Bortezomib, HB = HB 22.7. **n = 5 mice per group**

**Table 2:** Statistical analysis

Group Comparisons*	*Χ^2^*	P-Value
Bz *v*. Control	0.868	0.349
Bz → HB *v*. Control	1.044	0.307
**HB → Bz *v*. Control**	**3.884**	0.049
Bz → HB *v*. Bz	0.001	0.972
HB → Bz *v*. Bz	1.058	0.304
HB → Bz *v*. Bz →HB	1.324	0.250

* Statistical comparisons between groups were calculated as described in Materials and Methods. The arrow denotes the order of treatments. Bz = Bortezomib, HB = HB 22.7. **N = 5 mice per group; p < 0.05 is considered significant**.

In summary, the administration of HB22.7 followed by bortezomib is cytotoxic in an *in vitro *lymphoma cell culture model. This synergistic cytotoxicity is the result of, at least in part, enhanced apoptosis and increased ROS generation and is dependent upon the order of administration. Finally, the *in vitro *efficacy of HB22.7 followed by bortezomib was also seen in an *in vivo *xenograft model with no corresponding increase in toxicity. The sequence dependent synergy of the two drugs may be due to a priming effect of HB22.7 which would render cells more sensitive to bortezomib. Studies to determine the mechanism are ongoing. Nevertheless, clinical trails assessing the impact of sequencing of mAbs with bortezomib should be undertaken to determine the optimal efficacy of the combination.

## Abbreviations

NHL: non-Hodgkin's lymphoma; mAb: monoclonal antibody; ROS: reactive oxygen species; MCL: mantle cell lymphoma; FL: follicular lymphoma.

## Competing interests

Eric Churchill is an employee of Millennium Pharmaceuticals. All other authors declare that they have no competing interests.

## Authors' contributions

SM generated the figures and tables. Preformed the calculations and statistical analysis, drafted the manuscript and participated in editing the manuscript. EC generated figures and tables, preformed calculations and statistical analysis, helped to draft the initial draft of the manuscript and participated in editing the manuscript. YM carried out the animal studies. HM and CM carried out the *in vitro *cytotoxicity and ROS assays, and flow cytometric analysis. RO and JT conceived of the study, designed the experiments, participated in the data analysis, edited and finalized the manuscript. All authors read and approved the final manuscript.

## References

[B1] Schumer ST Joyce RM Radioimmunotherapy for Non-Hodgkin's Lymphoma Progress in Oncology 2003 46 72

[B2] Ries L Melbert D Krapcho M Stinchcomb D Howlader N Horner M Mariotto A Miller B Feuer E Altekruse S Lewis D Clegg L Eisner M Reichman M Edwards Be SEER Cancer Statistics Review, 1975-2005 2008 National Cancer Institute, Bethesda, MD

[B3] Voorhees PM Orlowski RZ The proteasome and proteasome inhibitors in cancer therapy Annu Rev Pharmacol Toxicol 2006 46 189 213 10.1146/annurev.pharmtox.46.120604.141300 16402903

[B4] Hideshima T Richardson P Chauhan D Palombella VJ Elliott PJ Adams J Anderson KC The proteasome inhibitor PS-341 inhibits growth, induces apoptosis, and overcomes drug resistance in human multiple myeloma cells Cancer Res 2001 61 3071 3076 11306489

[B5] Perez-Galan P Roue G Villamor N Montserrat E Campo E Colomer D The proteasome inhibitor bortezomib induces apoptosis in mantle-cell lymphoma through generation of ROS and Noxa activation independent of p53 status Blood 2006 107 257 264 10.1182/blood-2005-05-2091 16166592

[B6] Fribley A Zeng Q Wang CY Proteasome inhibitor PS-341 induces apoptosis through induction of endoplasmic reticulum stress-reactive oxygen species in head and neck squamous cell carcinoma cells Mol Cell Biol 2004 24 9695 9704 10.1128/MCB.24.22.9695-9704.2004 15509775 PMC525474

[B7] Obeng EA Carlson LM Gutman DM Harrington WJ Jr Lee KP Boise LH Proteasome inhibitors induce a terminal unfolded protein response in multiple myeloma cells Blood 2006 107 4907 4916 10.1182/blood-2005-08-3531 16507771 PMC1895817

[B8] Dong H Chen L Chen X Gu H Gao G Gao Y Dong B Dysregulation of unfolded protein response partially underlies proapoptotic activity of bortezomib in multiple myeloma cells Leuk Lymphoma 2009 50 974 984 10.1080/10428190902895780 19391038

[B9] Pham LV Tamayo AT Yoshimura LC Lo P Ford RJ Inhibition of constitutive NF-kappa B activation in mantle cell lymphoma B cells leads to induction of cell cycle arrest and apoptosis J Immunol 2003 171 88 95 12816986 10.4049/jimmunol.171.1.88

[B10] Fu DX Tanhehco Y Chen J Foss CA Fox JJ Chong JM Hobbs RF Fukayama M Sgouros G Kowalski J Pomper MG Ambinder RF Bortezomib-induced enzyme-targeted radiation therapy in herpesvirus-associated tumors Nat Med 2008 14 1118 1122 10.1038/nm.1864 18776891 PMC2709824

[B11] Goy A Younes A McLaughlin P Pro B Romaguera JE Hagemeister F Fayad L Dang NH Samaniego F Wang M Broglio K Samuels B Gilles F Sarris AH Hart S Trehu E Schenkein D Cabanillas F Rodriguez AM Phase II study of proteasome inhibitor bortezomib in relapsed or refractory B-cell non-Hodgkin's lymphoma J Clin Oncol 2005 23 667 675 10.1200/JCO.2005.03.108 15613697

[B12] Belch A Kouroukis CT Crump M Sehn L Gascoyne RD Klasa R Powers J Wright J Eisenhauer EA A phase II study of bortezomib in mantle cell lymphoma: the National Cancer Institute of Canada Clinical Trials Group trial IND.150 Ann Oncol 2007 18 116 121 16971665 10.1093/annonc/mdl316

[B13] Fisher RI Bernstein SH Kahl BS Djulbegovic B Robertson MJ de Vos S Epner E Krishnan A Leonard JP Lonial S Stadtmauer EA O'Connor OA Shi H Boral AL Goy A Multicenter phase II study of bortezomib in patients with relapsed or refractory mantle cell lymphoma J Clin Oncol 2006 24 4867 4874 10.1200/JCO.2006.07.9665 17001068

[B14] Strauss SJ Maharaj L Hoare S Johnson PW Radford JA Vinnecombe S Millard L Rohatiner A Boral A Trehu E Schenkein D Balkwill F Joel SP Lister TA Bortezomib therapy in patients with relapsed or refractory lymphoma: potential correlation of in vitro sensitivity and tumor necrosis factor alpha response with clinical activity J Clin Oncol 2006 24 2105 2112 10.1200/JCO.2005.04.6789 16606971

[B15] O'Connor OA Wright J Moskowitz C Muzzy J MacGregor-Cortelli B Stubblefield M Straus D Portlock C Hamlin P Choi E Dumetrescu O Esseltine D Trehu E Adams J Schenkein D Zelenetz AD Phase II clinical experience with the novel proteasome inhibitor bortezomib in patients with indolent non-Hodgkin's lymphoma and mantle cell lymphoma J Clin Oncol 2005 23 676 684 10.1200/JCO.2005.02.050 15613699

[B16] de Vos S Goy A Dakhil SR Saleh MN McLaughlin P Belt R Flowers CR Knapp M Hart L Patel-Donnelly D Glenn M Gregory SA Holladay C Zhang T Boral AL Multicenter randomized phase II study of weekly or twice-weekly bortezomib plus rituximab in patients with relapsed or refractory follicular or marginal-zone B-cell lymphoma J Clin Oncol 2009 27 5023 5030 10.1200/JCO.2008.17.7980 19770386

[B17] Tuscano J Engel P Tedder TF Kehrl JH Engagement of the adhesion receptor CD22 triggers a potent stimulatory signal for B cells and blocking CD22/CD22L interactions impairs T-cell proliferation Blood 1996 87 4723 4730 8639842

[B18] Tedder TF Tuscano J Sato S Kehrl JH CD22, a B lymphocyte-specific adhesion molecule that regulates antigen receptor signaling Annu Rev Immunol 1997 15 481 504 10.1146/annurev.immunol.15.1.481 9143697

[B19] Sato S Tuscano JM Inaoki M Tedder TF CD22 negatively and positively regulates signal transduction through the B lymphocyte antigen receptor Semin Immunol 1998 10 287 297 10.1006/smim.1998.0121 9695185

[B20] Tuscano JM Riva A Toscano SN Tedder TF Kehrl JH CD22 cross-linking generates B-cell antigen receptor-independent signals that activate the JNK/SAPK signaling cascade Blood 1999 94 1382 1392 10438726

[B21] Haas KM Sen S Sanford IG Miller AS Poe JC Tedder TF CD22 ligand binding regulates normal and malignant B lymphocyte survival in vivo J Immunol 2006 177 3063 3073 16920943 10.4049/jimmunol.177.5.3063

[B22] Tuscano JM O'Donnell RT Miers LA Kroger LA Kukis DL Lamborn KR Tedder TF DeNardo GL Anti-CD22 ligand-blocking antibody HB22.7 has independent lymphomacidal properties and augments the efficacy of 90Y-DOTA-peptide-Lym-1 in lymphoma xenografts Blood 2003 101 3641 3647 10.1182/blood-2002-08-2629 12511412

[B23] Bhalla S Balasubramanian S David K Sirisawad M Buggy J Mauro L Prachand S Miller R Gordon LI Evens AM PCI-24781 induces caspase and reactive oxygen species-dependent apoptosis through NF-kappaB mechanisms and is synergistic with bortezomib in lymphoma cells Clin Cancer Res 2009 15 3354 3365 10.1158/1078-0432.CCR-08-2365 19417023 PMC2704489

[B24] Haritunians T Mori A O'Kelly J Luong QT Giles FJ Koeffler HP Antiproliferative activity of RAD001 (everolimus) as a single agent and combined with other agents in mantle cell lymphoma Leukemia 2007 21 333 339 10.1038/sj.leu.2404471 17136116

[B25] Smolewski P Duechler M Linke A Cebula B Grzybowska-Izydorczyk O Shehata M Robak T Additive cytotoxic effect of bortezomib in combination with anti-CD20 or anti-CD52 monoclonal antibodies on chronic lymphocytic leukemia cells Leuk Res 2006 30 1521 1529 10.1016/j.leukres.2006.03.005 16630656

[B26] Wang M Han XH Zhang L Yang J Qian JF Shi YK Kwak LW Romaguera J Yi Q Bortezomib is synergistic with rituximab and cyclophosphamide in inducing apoptosis of mantle cell lymphoma cells in vitro and in vivo Leukemia 2008 22 179 185 10.1038/sj.leu.2404959 17898787

[B27] Alinari L White VL Earl CT Ryan TP Johnston JS Dalton JT Ferketich AK Lai R Lucas DM Porcu P Blum KA Byrd JC Baiocchi RA Combination bortezomib and rituximab treatment affects multiple survival and death pathways to promote apoptosis in mantle cell lymphoma MAbs 2009 1 31 40 10.4161/mabs.1.1.7472 20046572 PMC2715189

[B28] Jazirehi AR Gan XH De Vos S Emmanouilides C Bonavida B Rituximab (anti-CD20) selectively modifies Bcl-xL and apoptosis protease activating factor-1 (Apaf-1) expression and sensitizes human non-Hodgkin's lymphoma B cell lines to paclitaxel-induced apoptosis Mol Cancer Ther 2003 2 1183 1193 14617792

[B29] Jazirehi AR Huerta-Yepez S Cheng G Bonavida B Rituximab (chimeric anti-CD20 monoclonal antibody) inhibits the constitutive nuclear factor-{kappa}B signaling pathway in non-Hodgkin's lymphoma B-cell lines: role in sensitization to chemotherapeutic drug-induced apoptosis Cancer Res 2005 65 264 276 15665303

[B30] Hinz M Krappmann D Eichten A Heder A Scheidereit C Strauss M NF-kappaB function in growth control: regulation of cyclin D1 expression and G0/G1-to-S-phase transition Mol Cell Biol 1999 19 2690 2698 10082535 10.1128/mcb.19.4.2690 PMC84062

[B31] Bogner C Ringshausen I Schneller F Fend F Quintanilla-Martinez L Hacker G Goetze K Oostendorp R Peschel C Decker T Inhibition of the proteasome induces cell cycle arrest and apoptosis in mantle cell lymphoma cells Br J Haematol 2003 122 260 268 10.1046/j.1365-2141.2003.04438.x 12846895

[B32] Bellosillo B Villamor N Lopez-Guillermo A Marce S Esteve J Campo E Colomer D Montserrat E Complement-mediated cell death induced by rituximab in B-cell lymphoproliferative disorders is mediated in vitro by a caspase-independent mechanism involving the generation of reactive oxygen species Blood 2001 98 2771 2777 10.1182/blood.V98.9.2771 11675350

[B33] Shah MA Schwartz GK The relevance of drug sequence in combination chemotherapy Drug Resist Updat 2000 3 335 356 10.1054/drup.2000.0165 11498402

[B34] Smorenburg CH Sparreboom A Bontenbal M Verweij J Combination chemotherapy of the taxanes and antimetabolites: its use and limitations Eur J Cancer 2001 37 2310 2323 10.1016/S0959-8049(01)00309-4 11720823

[B35] Guichard S Cussac D Hennebelle I Bugat R Canal P Sequence-dependent activity of the irinotecan-5FU combination in human colon-cancer model HT-29 in vitro and in vivo Int J Cancer 1997 73 729 734 10.1002/(SICI)1097-0215(19971127)73:5<729::AID-IJC20>3.0.CO;2-# 9398054

[B36] Aissat N Le Tourneau C Ghoul A Serova M Bieche I Lokiec F Raymond E Faivre S Antiproliferative effects of rapamycin as a single agent and in combination with carboplatin and paclitaxel in head and neck cancer cell lines Cancer Chemother Pharmacol 2008 62 305 313 10.1007/s00280-007-0609-2 17912526

[B37] Fujimoto S Chikazawa H Schedule-dependent and -independent antitumor activity of paclitaxel-based combination chemotherapy against M-109 murine lung carcinoma in vivo Jpn J Cancer Res 1998 89 1343 1351 10.1111/j.1349-7006.1998.tb00532.x 10081496 PMC5921735

[B38] Adel AL Dorr RT Liddil JD The effect of anticancer drug sequence in experimental combination chemotherapy Cancer Invest 1993 11 15 24 10.3109/07357909309020256 7678539

[B39] Bonvini P Zorzi E Basso G Rosolen A Bortezomib-mediated 26S proteasome inhibition causes cell-cycle arrest and induces apoptosis in CD-30+ anaplastic large cell lymphoma Leukemia 2007 21 838 842 17268529 10.1038/sj.leu.2404528

[B40] Zheng B Georgakis GV Li Y Bharti A McConkey D Aggarwal BB Younes A Induction of cell cycle arrest and apoptosis by the proteasome inhibitor PS-341 in Hodgkin disease cell lines is independent of inhibitor of nuclear factor-kappaB mutations or activation of the CD30, CD40, and RANK receptors Clin Cancer Res 2004 10 3207 3215 10.1158/1078-0432.CCR-03-0494 15131062

[B41] Nencioni A Hua F Dillon CP Yokoo R Scheiermann C Cardone MH Barbieri E Rocco I Garuti A Wesselborg S Belka C Brossart P Patrone F Ballestrero A Evidence for a protective role of Mcl-1 in proteasome inhibitor-induced apoptosis Blood 2005 105 3255 3262 10.1182/blood-2004-10-3984 15613543

[B42] Herrant M Jacquel A Marchetti S Belhacene N Colosetti P Luciano F Auberger P Cleavage of Mcl-1 by caspases impaired its ability to counteract Bim-induced apoptosis Oncogene 2004 23 7863 7873 10.1038/sj.onc.1208069 15378010

[B43] Chaouchi N Vazquez A Galanaud P Leprince C B cell antigen receptor-mediated apoptosis. Importance of accessory molecules CD19 and CD22, and of surface IgM cross-linking J Immunol 1995 154 3096 3104 7534787

